# Peptidoglycan as a Bioactive Functional Dietary Supplement Enhances Growth, Improves Nutrient Digestion, Increases Cellular Energy Allocation, and Activates Viral Resistance of Juvenile *Penaeus monodon*

**DOI:** 10.1155/ijm/5341732

**Published:** 2026-09-06

**Authors:** Maila V. Pan, Vyenge Erre D. Gayosa, Rex Ferdinand M. Traifalgar

**Affiliations:** ^1^ Institute of Aquaculture, College of Fisheries and Ocean Sciences, University of the Philippines Visayas, Miagao, Iloilo, Philippines; ^2^ College of Fisheries and Marine Sciences, Zamboanga State College of Marine Sciences and Technology, Fort Pilar, Zamboanga City, Zamboanga del Sur, Philippines; ^3^ Graduate School, University of the Philippines Visayas, Iloilo City, Iloilo, Philippines

**Keywords:** cellular energy allocation, digestibility, digestive enzymes, immune indices, white spot syndrome virus

## Abstract

Immunostimulants are widely used in aquaculture to prevent diseases and have also been observed to promote growth in aquatic animals. The current study investigated the growth‐promoting mechanism of dietary peptidoglycan (PG) in black tiger shrimp (*Penaeus monodon*). PG was supplemented into the experimental diets at varying concentrations (0.00, 0.10, 0.20, and 0.40 g·kg^−1^ of feed) and administered to the shrimp over a 60‐day feeding trial. Shrimp growth in terms of weight gain and specific growth rate, total feed intake, feed conversion efficiency, protein efficiency ratio, and survival was monitored during the feeding trial. Immune parameters and shrimp viral resistance against white spot syndrome virus (WSSV) were also assessed. Results showed that supplementation of 0.20 g PG kg^−1^ diet enhanced shrimp growth compared with the control. This is supported by the biochemical analyses (nutrient retention, feed digestibility, digestive enzyme activities, and cellular energy allocation) showing improved feed conversion efficiency and lipid retention resulting from improved apparent dry matter digestibility of the diet, enzyme activities, and energy allocation in shrimp provided with 0.20 g PG kg^−1^ diet compared with the control. Furthermore, the 0.20‐g PG kg^−1^ diet also resulted in the highest survival rate in the WSSV challenge test compared with the control and other PG‐treated diets. This enhanced resistance was associated with an elevated total hemocyte count, increased respiratory burst activity, and increased phenoloxidase activity compared with the control. Collectively, the present data suggest that immunostimulant supplementation at 0.20 g PG kg^−1^ diet has growth‐promoting effects in juvenile *P. monodon*.

## 1. Introduction

Immunostimulants are substances such as *β*‐glucans, bacterial products (peptidoglycans and lipopolysaccharides), and plant constituents [1–4] that enhance the nonspecific defense mechanism and immune responses to provide resistance against potentially invasive, disease‐causing organisms or pathogens [3, 4] during periods of high stress [1]. It has been widely administered in cultured penaeids and found that these immunostimulants enhanced immune response against pathogens (ex. *Vibrio* sp., white spot syndrome virus [WSSV], and *Fusarium* sp.), resulting in high survival as well as enhanced growth [5–9].

Peptidoglycan, a component of the bacterial cell wall, was found to have growth‐promoting factors on *Penaeus monodon*, in addition to its immunostimulating properties [10]. Itami et al. [11] observed that peptidoglycan‐fed kuruma prawn (*Marsupenaeus japonicus*) exhibited higher growth rates than nonimmunostimulated shrimp. Other immunostimulants such as *β*‐glucan were also found to enhance the growth of shrimp, given singly or in combination with vitamin C [11–13]. Wu et al. [14] have also shown that the growth of *P. monodon* was enhanced by the application of 500–2000 mg fucoidan kg^−1^ diet from wakame (*Undaria pinnatifida*).

The effects of immunostimulants on the growth of finfishes and crustaceans have been observed to influence weight gain, feed conversion ratio, nutrient retention, and digestive enzyme activities. Despite these studies, it has not been clearly defined how these substances induce growth in shrimp, how they affect digestibility, and how they influence metabolism. There is still a need to investigate further the mechanism of how immunostimulants affect the growth performance of penaeids. Thus, an experiment was conducted to elucidate the mechanism behind this phenomenon. Eissa et al. [9] reported that the growth‐promoting effects of peptidoglycan were due to an increase in muscle growth and enhanced digestive protease activities. However, the said study was conducted for only 30 days, and indices were only evaluated.

To provide a more comprehensive understanding on the growth‐promoting mechanisms of immunostimulants, this more extensive study was carried out. The present study would further elucidate the growth‐promoting effects of dietary peptidoglycan in juvenile *P. monodon* through monitoring its growth, nutrient retention, diet digestibility, digestive enzyme activities, and cellular energy allocation (CEA). To our knowledge, this is the first study to elucidate the mechanisms underlying the growth‐promoting effects of dietary peptidoglycan in shrimp while simultaneously demonstrating its immunostimulatory benefits.

## 2. Materials and Methods

### 2.1. Ethical Approval

Animals used in this study were handled carefully and humanely. Animals sampled for the different analyses were anesthetized by lowering the temperature. Applicable ethical guidelines were followed during the conduct of the experiments.

### 2.2. Experimental Animal and Diet Preparation

WSSV‐free *P. monodon* postlarvae (*PL12*) were purchased from a local hatchery in Tigbauan, Iloilo, Philippines, and transported to the Institute of Aquaculture Multispecies Hatchery, College of Fisheries and Ocean Sciences, University of the Philippines Visayas, Miagao, Iloilo, Philippines. The shrimp were acclimated in a recirculating setup and were given a commercial diet for 2 weeks before the start of the experiment. The physicochemical water parameters were regularly monitored and maintained at the optimum (salinity: 22–25 ppt, dissolved oxygen: 11–12.5 ppm, temperature: 26.1–29°C, pH: 8.1–8.4, ammonia: < 0.1 ppm). Commercially available peptidoglycan (Chemoforma Ltd., Augst, Switzerland) was used and included in the formulated diets at the following concentrations: 0.10, 0.20, and 0.40 g PG kg^−1^ diet. These concentrations were based on the results of Purivirojkul et al. [15], wherein they also used peptidoglycan in *P. monodon*, focusing on its immune response. This is a more intensive study of what has been reported by Eissa et al. [9], wherein the present study elucidates further the mechanisms of the growth promotion in shrimp peptidoglycan, which was also found as a consequence of the enhancement of its immune response. The basal diet (Table 1) was formulated according to Deshimaru et al. [16] to meet the *P. monodon*′s nutrient requirement. Peptidoglycan was incorporated into the experimental diets by proportionally reducing the cellulose content to maintain a constant total diet weight and ensure consistent nutrient composition across all dietary treatments. The basal diet was submitted to the Department of Science and Technology–Regional Standards and Testing Laboratory for proximate composition analyses.

**Table 1:** Formulation and nutrient composition of the basal diet.

Ingredients	g 100 g^−1^ (% dry weight)
Peruvian fishmeal	20.00
Shrimp meal	27.00
Gelatin	5.00
Soybean meal	20.00
Starch	15.00
Vitamin mix^a^	1.75
Mineral mix^b^	1.75
Cod liver oil	4.00
Soy lecithin	0.50
Butylated hydroxytoluene (BHT)	0.02
Cellulose	4.98
Peptidoglycan	0.00

**Proximate composition**	

Crude protein	42.28 ± 0.11
Crude fat	9.12 ± 0.16
Ash	15.43 ± 0.07
Crude fiber	7.28 ± 0.17
Nitrogen‐free extract	25.89 ± 0.35

^a^Vitamin premix contributed the following per g of feed: *Β*‐carotene, 36 *μ*g·g^−1^; cholecalciferol, 3 *μ*g·g^−1^; thiamin, 72 *μ*g·g^−1^; riboflavin, 144 *μ*g·g^−1^; pyridoxine, 132 *μ*g·g^−1^; cyanocobalamin, 0.4 *μ*g·g^−1^; a‐tocopherol, 330 *μ*g·g^−1^; menadione, 48 *μ*g·g^−1^; niacin, 288 *μ*g·g^−1^; pantothenic acid, 80 *μ*g·g^−1^; biotin, 0.4 *μ*g·g^−1^; folic acid, 24 *μ*g·g^−1^; inositol, 600 *μ*g·g^−1^; and stay C, 2000 *μ*g·g^−1^.

^b^Mineral premix contributed the following per g of feed: P, 2400 *μ*g·g^−1^; Ca, 2400 *μ*g·g^−1^; Mg, 300 *μ*g·g^−1^; Fe, 30 *μ*g·g^−1^; Zn, 84 *μ*g·g^−1^; Cu, 42 *μ*g·g^−1^; K, 1500 *μ*g·g^−1^; Co, 22 *μ*g·g^−1^; Mn, 32 *μ*g·g^−1^; Se, 0.02 *μ*g·g^−1^; Mo, 0.01 *μ*g·g^−1^; Al, 0.5 *μ*g·g^−1^; I, 8 *μ*g·g^−1^.

### 2.3. Growth Performance Evaluation

*P. monodon* postlarvae with an initial weight of 0.01 ± 0.00 g were randomly distributed to the 50‐L experimental aquaria at a stocking density of 25 postlarvae per aquarium, constituting four treatments replicated three times in a completely randomized design. The diets were given to the experimental animals at a 15% feeding rate based on the average body weight, four times a day (08:00, 11:00, 14:00, and 17:00 h) for 60 days. Biomass sampling was done every 15 days to assess shrimp weight and adjust feed allocation. Biological parameters such as percent weight gain, specific growth rate (SGR), feed conversion efficiency (FCE), protein efficiency ratio (PER), survival, and nutrient retention were determined according to Deshimaru et al. [16]. At the end of the growth trial, shrimp samples were collected randomly from the control and the best treatment group, stored at −80°C, and subsequently used in various analyses, such as immune assays, digestive enzyme activity, and CEA assays. Experimental animals for the WSSV challenge test were also allocated.

### 2.4. Digestive Enzyme Assays

The digestive health of *P. monodon* was evaluated in terms of total protease, amylase, and lipase activities. Crude enzyme extracts used in the assays were prepared from shrimp hepatopancreas homogenized at a 1:25 ratio (wet tissue to volume) in a cold 50‐mM citrate‐phosphate buffer (pH 7.0) for amylase and lipase assays, whereas a 10‐mM sodium acetate buffer with 5 mM calcium acetate (pH 7.5) was used for the protease assays. Protein content of the extracts was quantified following the protocol of Halver and Hardy [17]. Total protease was measured following the methods of Folin and Ciocalteu [18] and Anson [19] using casein as a substrate. Buffered starch solution was used as a substrate for *α*‐amylase activity following the method of Bernfeld [20] as modified by Areekijseree et al. [21]. One unit of enzyme activity for protease and *α*‐amylase was expressed as product produced min^−1^ mg^−1^ protein. Lipase activity was analyzed following the methods of Borlongan [22] using olive oil as substrate. A unit of lipase activity was defined as the volume of 0.01 N NaOH required to neutralize the fatty acid released after a 1‐h incubation.

### 2.5. CEA Assay

The CEA assay was conducted according to Rueda‐Jasso et al. [23] and Verslycke et al. [24]. This assay was carried out in triplicate, using shrimp tail muscles as samples. The supernatant used in the assay was collected from the tail muscles homogenized in an ice‐cold buffer or ETS B (75 *μ*M MgSO_4_, 1.5 mg.mL^−1^ polyvinylpyrrolidone, and 0.2% [v: v] Triton X‐100 in 0.1 M phosphate buffer, pH 8.5). The total protein content of the samples was also determined according to Bradford [25]. Total carbohydrate content was analyzed following the protocol of Masuko et al. [26] using sulfuric acid and phenol, and measuring the absorbance at 490 nm. Total lipid was subsequently determined following the methods of Bligh and Dyer [27] with modifications, using chloroform and methanol. The energy consumed (Ec) was estimated by quantifying the electron transport system (ETS) activity following the protocols of Owens and King [28]. An aliquot of the cell‐free extract was diluted to ensure that the samples contained less than 5 mg wet weight (0.5 mg protein). Nexxt, 0.5 mL of the resulting cell‐free extract was incubated with 1.5 mL of substrate solution (1.7 mM nicotinamide adenine dinucleotide‐NADH, 0.25 mM nicotinamide adenine dinucleotide phosphate‐NADPH, and 0.2% [v:v] Triton X‐100 in 0.1 M phosphate buffer, pH 8.5) and 0.5 mL of tetrazolium salt 2‐(p‐iodophenyl)‐3‐(p nitrophenyl)‐5‐phenyl tetrazolium chloride (INT) solution (2 mg INT mL^−1^ in double distilled water) in a dark place at 25°C for 10 min. The final 2.5 mL reaction mixture contained the cell‐free extract, 15 *μ*M MgSO_4_, 0.3 mg mL^−1^ PVP, 1.0 mM NADH, 0.15 mM NADPH, 0.4 mg mL^−1^ INT, and 0.16% (v:v) Triton X‐100 in 0.08 M phosphate buffer, pH 8.5. The reaction was halted by the addition of 0.5 mL quench solution (50% formalin, 50% 1 M H_3_PO_4_, pH 2.5), making the final volume 3 mL. The sample absorbance was determined at 490 nm and corrected by a turbidity blank (0.5 mL cell‐free extract, 2 mL 0.1 M phosphate buffer, pH 8.5, 0.5 mL quench) and a substrate‐INT (abiotic) blank (0.5 mL ETS B, 1.5 mL substrate, 0.5 mL INT, incubated for 10 min and quenched). The INT‐formazan molar extinction coefficient in 0.133% Triton X‐100 of 15 900 M^−1^ cm^−1^ was used in the calculation of the amount of formazan formed. ETS activity was expressed in mol O_2_ g^−1^ wet weight and following equation:

Concentrationformazan=AbsrobanceExtinction coefficient×path length.

The various energy reserve fractions (Ea), which include protein, sugar, and lipid, were converted into energetic equivalents by applying their corresponding combustion energy (24 kJ g^−1^ protein, 17.5 kJ g^−1^ carbohydrates, and 39.5 kJ g^−1^ lipid) [29]. Using cellular respiration rate (ETS) data, the Ec was calculated using the stoichiometric relationship, which states that 1 mol of O_2_ is consumed for every 2 mol of formazan. The oxyenthalpic equivalents for an average protein, sugar, and lipid mixture are 484 kJ mol^−1^ O_2_, consumption into energy equivalents [29]. The Ea, Ec, and CEA values were computed as follows:

Available energy Ea=protein+sugar+lipidkJ/g wet weight.

Energy consumption Ec=ETS activitykJ/g wet weight.

Cellular Energy Allocation CEA=Ea−EckJ/g wet weight.

### 2.6. Immune Assays

Immune indices of shrimp were carried out in triplicate per treatment; hemolymph was collected according to the methods of Hernández‐López et al. [30]. Then, total hemocyte counts were counted in a Neubauer hemocytometer at 40× magnification after adding Rose Bengal solution. Hemocyte counts were expressed as total hemocyte count.mL^−1^ hemolymph [31]. Nitroblue tetrazolium reduction to insoluble blue formazan as a probe for superoxide anion production was used in quantifying the respiratory burst of hemocytes following the protocols of Muñoz et al. [32] with modifications. The absorbance was then determined at 620 nm in a LEDETECT 96‐microplate reader, and the activity was presented as optical density (OD) 100 *μ*L^−1^ hemolymph. Phenoloxidase activity was determined spectrophotometrically according to the method of Hernández‐López et al. [30] with modifications. The optical density was measured at 490 nm using a LEDETECT 96‐microplate reader every 30 s for 3 min. Enzyme activity was presented as the increase in absorbance min^−1^ 100 *μ*L^−1^ hemolymph according to Joseph and Philip [33]. One enzyme activity is equivalent to the increase of 0.001 in absorbance.

### 2.7. Digestibility Trial and Chromium Analysis

Shrimp were adapted to the experimental diets for 5 days before feces collection. The animals were maintained with diets containing 1% of chromic oxide mixed in the formulated diets. Similar to the WSSV challenge test and immune assays, only two treatments were run: the control and 0.20 g PG kg^−1^ diet groups, which exhibited the highest growth rate among the treatments. The experimental units used were 67‐L plastic tanks with triplicates. This is one of the limitations of this digestibility trial for this study. Feces were collected for 30 days until more than 30 g of feces (wet basis) were obtained. The shrimps (1.16 ± 0.10 g mean weight) were first fed at a biomass of 10%, and the ration was changed every day to minimize the amount of uneaten feed. Feces were collected six times a day according to the protocol of Cruz‐Suárez et al. [34]. The shrimp′s feces were collected using the following methods in successive tanks: at 06:30, the bottom of the first tank was siphoned to remove any uneaten feed and molts; at 07:00, the animals were fed; at 08:00, the remaining feed and the first feces sample was discarded; and at 09:00 and 10:15, the first and second feces collections were siphoned, immediately rinsed with distilled water, and stored in the freezer. In the afternoon, a similar procedure was carried out twice. Before being analyzed, the fecal wastes from a specific tank were pooled, frozen (−20°C), and oven‐dried. Processing of samples for chromium analyses was done in triplicate. Moisture and ash content of the fecal samples were determined [35]. The content of the samples was analyzed using flame atomic absorption spectrophotometry [36]. Apparent dry matter digestibility (ADMD) of the diets was computed according to Bautista‐Teruel et al. [37]:

ADMD %=100−100×CrdietDMdiet×DMfecesCrfeces,

where Cr—chromium and DM—dry matter.

### 2.8. Disease Resistance Trial

**Figure 1:**
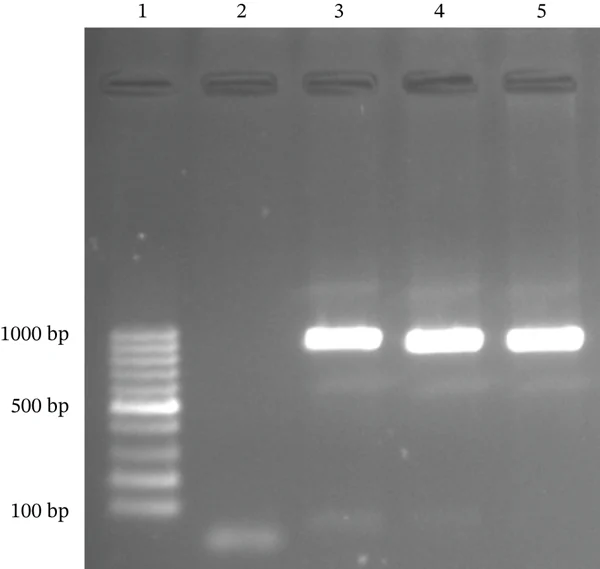
PCR result of WSSV. Lane 1—marker; Lane 2—no primer control; Lane 3—WSSV positive control; Lane 4—pooled *P. monodon* samples in the control group exhibiting white spots on carapace; and Lane 5—*P. monodon* samples in the treated (0.20 g kg^−1^ peptidoglycan) group exhibiting white spots on carapace. Band formed at 982 bp: WSSV (+); absence of band: WSSV (−). Molecular markers are 100, 500, and 1000 bp.

For the disease resistance trial, three treatment groups were evaluated. A negative control composed of shrimp provided with the control diet and no viral infection, a positive control group where the control diet was provided and viral infection was administered, and the 0.20‐g PG kg^−1^ diet‐treated group. The trial was done in triplicate groups with randomly stocked 25 shrimps per tank, stocked into 30‐L containers. The experimental system utilized static water conditions with ample aeration. Activation of the WSSV was achieved by feeding infected tissues derived from artificially infected shrimp to a separate group of uninfected shrimp. The challenge was conducted via oral administration, wherein test animals were fed infected tissue at 10% of their average body weight, following a modified protocol based on Joseph and Philip [33]. The infected tissues were finely minced and given three times within a day (08:00, 12:00, and 16:00) to ensure effective viral transmission. This dietary dose was previously optimized to elicit 50% mortality in infected animals. Mortality of test animals was monitored twice daily (08:00 and 17:00). Moribund and dead prawns were collected and stored at −80°C for polymerase chain reaction assay to confirm that the mortality was due to WSSV infection (Figure 1).

### 2.9. Statistical Analysis

Data were reported as mean ± standard error of the mean. Results of the growth trial were subjected to one‐way analysis of variance (ANOVA), whereas the differences between means were tested using the Tukey test at a 0.05 significance level. Meanwhile, observations on the WSSV challenge were analyzed using the chi‐square test. Data on the immune indices, nutrient retention, ADMD, digestive enzyme activity, and CEA assays were analyzed using the *t*‐test.

## 3. Results

The present study further elucidated the mechanisms of the growth‐promoting effects of immunostimulants such as peptidoglycan aside from its main function as immunostimulant.

### 3.1. Growth Performance and Survival

After a 60‐day feeding trial, shrimp fed with diets containing 0.20 g PG kg^−1^ diet exhibited significantly higher percent weight gain and SGR (Table 2). This was followed by the weight gain and SGR of shrimp given a 0.40‐g PG kg^−1^ diet. Lower weight gains and SGRs were observed in shrimp maintained with diet supplemented with 0.10 g PG kg^−1^ diet and the control. In terms of PER, supplementation of the 0.20 g PG kg^−1^ diet resulted in significantly higher results but was found to be statistically like shrimp in the 0.10 g PG kg^−1^ diet and control groups. Shrimp provided with a 0.40‐g PG kg^−1^ diet had the lowest PER. Furthermore, FCE is also significantly higher in 0.20 g PG kg^−1^ diet compared with other treatments. However, total feed intake and shrimp survival were not significantly different from each other.

**Table 2:** Growth performance: weight gain (WG, %), specific growth rate (SGR, % day^−1^), feed conversion efficiency (FCE, %), protein efficiency ratio (PER, %), feed intake (FI, g dry weight fish^−1^), and survival (SUR, %) of *P. monodon* fed with varying levels of dietary peptidoglycan.

	0.00 PG kg^−1^	0.10 PG kg^−1^	0.20 PG kg^−1^	0.40 PG kg^−1^
WG	1060.56 ± 41.06^a^	1034.12 ± 88.77^a^	1323.40 ± 26.52^b^	1158.77 ± 1.44^ab^
SGR	4.08 ± 0.06^a^	4.04 ± 0.13^a^	4.43 ± 0.03^b^	4.22 ± 0.00^ab^
FCE	39.96 ± 0.75^a^	39.78 ± 1.43^a^	45.69 ± 0.55^b^	39.43 ± 1.10^a^
PER	0.21 ± 0.00^ab^	0.19 ± 0.01^ab^	0.21 ± 0.00^bc^	0.19 ± 0.01^a^
FI	0.29 ± 0.00^a^	0.30 ± 0.01^a^	0.31 ± 0.00^a^	0.30 ± 0.01^a^
SUR	98.67 ± 1.33^a^	100.00 ± 0.00^a^	98.67 ± 1.33^a^	100.00 ± 0.00^a^

*Note:* Values are mean ± standard error of the mean. Within rows, means with different superscript letters are significantly different (*p* < 0.05).

### 3.2. Shrimp Carcass Nutrient Composition and Retention

Shrimp carcass protein compositions are similar in both the control and the group given with 0.20 g PG kg^−1^ diet (Table 3). However, lipid content of shrimp fed with 0.20 g PG kg^−1^ diet was significantly higher than the control group. Similar observations have been noted in the nutrient retention of shrimps. Protein retention in the control and 0.20 g PG kg^−1^ diet groups was not significantly different, but lipid retention is higher in the treated group (0.20 g PG kg^−1^ diet) (Table 4).

**Table 3:** Average macronutrient composition (% dry weight basis) of *P. monodon* fed with varying levels of dietary peptidoglycan.

Treatments	Protein	Lipid
Control	74. 81 ± 0.49^a^	14.089 ± 0.04^b^
0.20 g PG kg^−1^	75.03 ± 1.2^a^	16.918 ± 0.49^a^

*Note:* Values are mean ± standard error of the mean. Within columns, means with different superscript letters are significantly different (*p* < 0.05).

**Table 4:** Nutrient retention (%) of *P. monodon* fed with varying levels of dietary peptidoglycan.

Treatments	Protein retention	Lipid retention
Control	15.358 ± 0.28^a^	12.040 ± 0.23^b^
0.20 g PG kg^−1^	16.196 ± 0.19^a^	14.618 ± 0.18^a^

*Note:* Values are mean ± standard error of the mean. Within columns, different superscript letters indicate significant difference (*p* < 0.05).

### 3.3. Diet Digestibility and Digestive Enzymes

**Figure 2:**
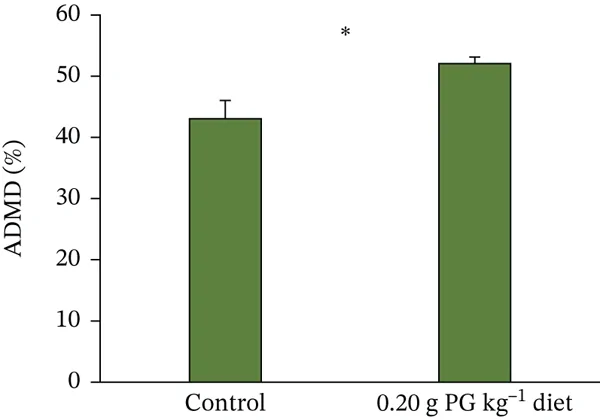
Apparent dry matter digestibility (ADMD) of *P. monodon* diets: control and 0.20 g PG kg^−1^ diet (mean ± SEM). Asterisk ( ^∗^) indicates significant difference among treatments (*p* < 0.05).

**Figure 3:**
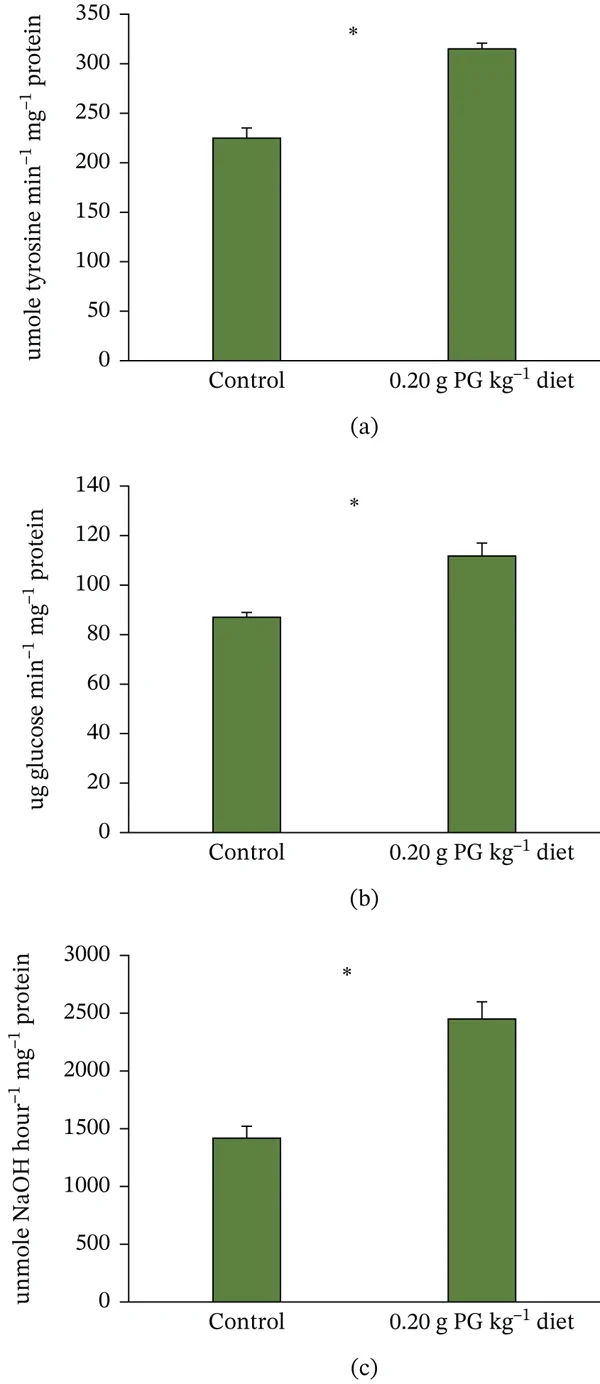
(a) Total protease, (b) *α*‐amylase, and (c) lipase activities of *P. monodon* (mean ± SEM). Asterisk ( ^∗^) indicates significant difference among treatments (*p* < 0.05).

Diet supplemented with 0.20 g PG kg^−1^, ADMD is significantly higher than the control group (Figure 2). This is also similar to the results in the digestive enzyme analyses in terms of protease, *α*‐amylase, and lipase wherein shrimp given 0.20 g PG kg^−1^ diet has significantly higher results than the shrimp in the control group (Figure 3).

### 3.4. CEA

Energy available which is the sum of protein, lipid, and carbohydrate contents of the shrimp tail muscles from the control and 0.20 g PG kg^−1^ diet is not significantly different (Table 5). However, Ec based on ETS assay in shrimp was lower in 0.20 g PG kg^−1^ diet than the control group. Similarly, CEA of shrimp is also higher in the treated group (0.20 g PG kg^−1^ diet) than the control group.

**Table 5:** Energetic content on the main macronutrients and energy consumed in the muscle of *P. monodon* fed with the control and 0.20 g PG kg^−1^ diet (kJ g^−1^ wet weight).

Parameters	0.00 g PG kg^−1^	0.20 g PG kg^−1^
Protein	3.26 ± 0.41^a^	3.93 ± 0.00^a^
Carbohydrates	0.054 ± 0.01^a^	0.046 ± 0.00^a^
Lipid	0.76 ± 0.05^a^	0.87 ± 0.10^a^
Energy available (E_a_)	4.08 ± 0.44^a^	4.85 ± 0.10^a^
Energy consumed (E_c_)	0.015 ± 0.00^b^	0.012 ± 0.00_a_
Cellular energy allocation (CEA)	4.06 ± 0.00^b^	4.83 ± 0.00^a^

*Note:* Values are mean ± standard error of the mean. Within rows, different superscript letters indicate significant difference (*p* < 0.05).

### 3.5. Immune Indices

Immune indices of shrimp in terms of total hemocyte count, respiratory burst, and phenoloxidase activity are significantly higher in the group given 0.20 g PG kg^−1^ diet than the control group (Table 6).

**Table 6:** Immune indices: total hemocyte count (THC), respiratory burst (RB) activity, and phenoloxidase (PO) activity of *P. monodon* fed with graded levels of dietary peptidoglycan.

	0.00 g PG kg^−1^	0.20 g PG kg^−1^
THC (cells mL^**−**1^)	334,074.07 ± 5604.72^b^	402,962.96 ± 2962.96^a^
RB (OD_620_ 100 *μ*L^**−**1^)	0.07 ± 0.00^b^	0.09 ± 0.00^a^
PO (*Δ*OD min^**−**1^ 100 *μ*L^**−**1^)	0.02 ± 0.00^b^	0.03 ± 0.00^a^

*Note:* Values are mean ± standard error. Within rows, different superscript letters indicate significant difference (*p* < 0.05).

### 3.6. WSSV Resistance

**Figure 4:**
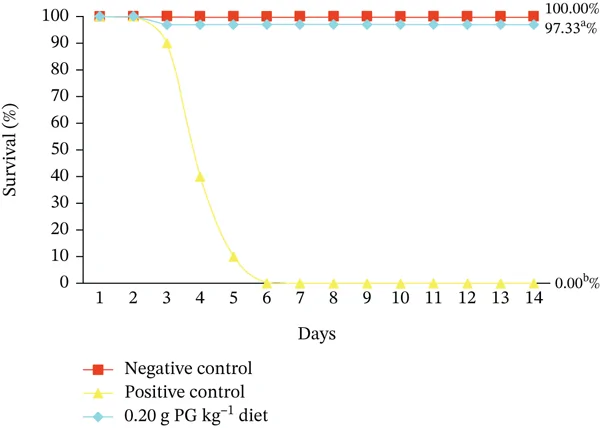
Survival of *P. monodon* fed with graded levels of peptidoglycan after 12 days of WSSV infection challenge. Different superscripts indicate significant difference (*p* < 0.05).

Shrimp given with 0.20 g PG kg^−1^ diet resulted in 97.33% survival upon exposure to WSSV, whereas the positive control group exposed to WSSV resulted in 100% mortality (Figure 4). The negative control group with no viral exposure showed 100% survival.

## 4. Discussion

The supplementation of 0.20 g PG kg^−1^ diet enhanced the growth of the *P. monodon* due to improved feed efficiency. This observation was due to its heightened immune response that was confirmed through its high survival against WSSV. Furthermore, aside from the good health status of shrimp, growth improvement was also due to better lipid retention. This better lipid retention in shrimp resulted from improved ADMD of the diet, digestive enzyme activities, and CEA.

Immunostimulants primarily boost the immune responses of various organisms; however, it was observed to be coupled by growth enhancement. This property of immunostimulants was confirmed in this study wherein *P. monodon* supplemented by 0.20 g PG kg^−1^ diet exhibited significantly higher SGR, better FCE, and increased lipid retention than the other treatment and control groups. These results imply that the said level of peptidoglycan in the diet is enough to enhance the growth of *P. monodon*. Survival in all treatments was not significantly different suggesting that the diets with peptidoglycan are acceptable and not toxic to shrimp postlarvae. The present findings agree with the results of several researchers upon feeding peptidoglycan‐supplemented diets to shrimp [10, 11, 38]. Several studies have reported on the growth‐promoting effects of shrimp, but limited information is available and has not been fully elucidated, which these studies would further explain.

The supplementation of 0.20 g PG kg^−1^ diet has been proven to enhance the survival of *P. monodon* against WSSV infection. This result confirms that peptidoglycan at this level has an immunoprotective effect on *P. monodon* against WSSV. Results of the present study conform to the earlier reports on the efficacy of peptidoglycan as immunostimulant on shrimps. Similar observations have been noted in shrimps fed with peptidoglycan supplemented diets and challenged with various pathogens [10, 11, 15, 38]. This immunoprotective effect of peptidoglycan on *P. monodon* at an optimum dose could be attributed to the enhancement of immune responses wherein 0.20 g PG kg^−1^ diet‐fed shrimp exhibited the highest total hemocyte count, respiratory burst, and phenoloxidase activities. Hemocytes serve a significant and central function in host defense wherein they are usually involved in phagocytosis, encapsulation, melanization, and coagulation [39]. Perazzolo et al. [40] stated that hemocyte number is sometimes used as an indicator of shrimp health status. Amar et al. [38] further added that increase in respiratory burst activity correlates to higher phagocytic activity. This conforms with the findings that the supplementation of peptidoglycan enhances survival of an organism against diseases was due to phagocytic activity [11, 41]. In addition, hemocytic, lysozyme and serum antibacterial activities might contribute to the disease resistance of aquatic organisms when fed with immunostimulants [7, 9, 15, 41]. Enhancement of the disease resistance in shrimp can also be attributed to the upregulation of immune‐related genes such as antilipopolysaccharide factor, crustin, peroxiredoxin (Prx), prophenoloxidase, and hemocyanin subunit L5 [2, 3, 42].

The significantly higher lipid retention of shrimps maintained with 0.20 g PG kg^−1^ diet compared with the control group implies that lipid retention was influenced by peptidoglycan supplementation but not the protein retention that was found not significant among treatments. This observation coincides with the previous observation of Pan et al. [10] on the increase in lipid retention of shrimp fed with dietary peptidoglycan. It can be stated that the growth of *P. monodon* is gearing towards fat deposition that may be due to the induction of the expression of immune cytokines such as tumor necrosis factor‐alpha (TNF‐*α*). This cytokine is involved in various biological functions that include inflammation, apoptosis, stimulation of immune responses and lipid metabolism [43–45]. Earlier reports stated that in invertebrates, TNF‐*α* is induced by peptidoglycan [43, 46]. Mekata et al. [47] reported the first conclusive evidence of the presence of TNF ligands in shrimps particularly *M. japonicus.* Furthermore, the TNF gene was also discovered by Wang et al. [48] from *L. vannamei*. It was stated that peptidoglycan enhanced the expression of the *MjTNF* gene in the lymphoid organ cells of *M. japonicus* and was also stimulated by lipopolysaccharides and *Vibrio penaeicida* [47]. Wang et al. [49] also found that the expression of this gene is influenced by *Vibrio alginolyticus*, *Staphylococcus aureus*, *Candida albicans*, and WSSV. In addition, Gioacchini et al. [50] also observed that the expression of liver cytokines that includes TNF‐*α* was stimulated by the administration of Ergosan in rainbow trout.

Another aspect investigated in this study is the effect of the digestibility of diets on the growth of peptidoglycan‐supplemented shrimps. The present findings show that supplementation of 0.20 g PG kg^−1^ diet enhanced the ADMD of the diets, which implies that there is efficient nutrient absorption that may have a positive effect on the enhancement of growth. This increase in the digestibility of the diets might be attributed to the significant enhancement of the digestive enzyme activities such as the total protease, *α*‐amylase, and lipase in comparison with the control. These results imply that the activities of these enzymes were enhanced by peptidoglycan that may be due to the stimulation of hepatopancreatic R‐cells, which are involved in the digestion and absorption of nutrients. R‐cells also function in the storage and metabolization of lipid droplets and glycogen [51, 52], which may also be related to the significant lipid retention observed in *P. monodon* in this study. Moreover, the increase in the digestive enzyme activity in shrimp might be also due to the increase in the number of B‐cells, with secretory granules, in the hepatopancreas of shrimp [42]. CEA assay has been widely utilized to assess the metabolic state of an organism in toxicity studies such as invertebrates [24, 53–58]. It was observed that CEA lowers upon exposure to stress or toxins. The result of the present study indicates that dietary peptidoglycan at 0.20 g PG kg^−1^ diet is not toxic to *P. monodon* since it has no adverse effects on the metabolic processes of the organism based on its higher CEA than the control group. This further confirms that the cellular condition of the organism is in good state.

At all life stages, organisms have limited energy budgets and thus allocate resources among competing demands for maintenance, growth, reproduction, and storage [59]. Steyermark [60] also stated that a trade‐off may exist between production (growth) and maintenance metabolism. The principle of allocation suggests that individuals with lower metabolic expenditures may have higher growth rates, whereas individuals with higher standard metabolic rates tend to have low growth rates. This principle shows a negative correlation between maintenance metabolism and growth. The Ec by the group maintained with 0.20 g PG kg^−1^ diet was lower, which implies that this treated group had lower energy consumption. This may be due to the much better condition of the peptidoglycan supplemented shrimp, and it does not need much energy to maintain the integrity of its cells and health status due to the activation of its immune responses. It also does not require much energy in assimilation of its nutrients in the diets due to the increase in its digestive enzyme activities. These results agree with the hypothesis of Traifalgar et al. [7] that the enhancement of metamorphic survival of *M. japonicus* larvae fed with fucoidan as immunostimulant could be due to the efficient utilization of cellular energy reserves. The low energy consumption and higher CEA explain the significantly better growth performance and nutrient retention of shrimp fed the peptidoglycan diet. This higher CEA in 0.20 g PG kg^−1^ diet‐fed shrimp also means that more energy is allocated for growth. This result is highly correlated with the weight gain, SGR, FCE, and lipid retention of the treated (0.20 g PG kg^−1^ diet) group. This is similar to the results obtained by [23] wherein they used CEA assay in assessing the effects of different dietary nonprotein energy levels in Senegalese sole (*Solea senegalensis* Kaup, 1858). They found out that a fish diet with 11% lipid and raw carbohydrate as an energy source exhibited the highest CEA among the treatments that means more energy allocated for growth. Their CEA results correlate with the individual weight, total biomass and feed conversion ratio of the experimental animal.

The findings of this study would help the aquaculture industry and scientific community by providing additional information on the mechanism of the growth‐promoting effects of immunostimulants, specifically dietary peptidoglycan on *P. monodon*, which might open opportunities for the application of various interventions on disease management.

## 5. Conclusion

Dietary supplementation with 0.20 g PG kg^−1^ increased weight gain by more than 200%, improved FCE from 39.96% to 45.69%, enhanced CEA from 4.06 to 4.83 kJ g^−1^ wet weight, and significantly improved survival following WSSV challenge by 97.33% compared with the control group. These findings demonstrate that dietary PG enhances nutrient utilization and CEA while maintaining its immunostimulatory activity, supporting the hypothesis that improved immune status exerts an energy‐sparing effect that allows more energy to be allocated towards growth. This study provides the first mechanistic evidence linking dietary PG supplementation with improved digestive physiology, energy allocation, and growth performance in *P. monodon*. Collectively, these findings highlight the potential of PG as a functional feed additive to improve shrimp production and disease resilience. Future studies should evaluate its long‐term efficacy under commercial farming conditions and further elucidate the molecular mechanisms underlying its effects on energy metabolism and immune regulation.

## Funding

This study was supported by Philippine Council for Agriculture, Aquatic and Natural Resources Research and Development.

## Conflicts of Interest

The authors declare no conflicts of interest.

## Data Availability

The data that support the findings of this study are available from the corresponding author upon reasonable request.
